# Proteomic analysis of mTOR inhibition-mediated phosphorylation changes in ribosomal proteins and eukaryotic translation initiation factors

**DOI:** 10.1007/s13238-016-0279-0

**Published:** 2016-06-08

**Authors:** Xu Jiang, Shan Feng, Yuling Chen, Yun Feng, Haiteng Deng

**Affiliations:** MOE Key Laboratory of Bioinformatics, School of Life Sciences, Tsinghua University, Beijing, 100084 China

**Dear Editor,**

The mammalian target of rapamycin (mTOR), as a critical energy sensor and cell-growth regulator, controls protein synthesis, autophagy and many important cellular processes through forming functional distinct complexes, mTORC1 and mTORC2. mTORC1 that is sensitive to rapamycin, regulates cell growth and protein synthesis, while mTORC2 that is insensitive to rapamycin, regulates cellular metabolism and the cytoskeletal organization (Gingras et al., 2001; Hay and Sonenberg, 2004). Translation initiation is the rate-limiting step in protein synthesis, which proceeds through a multi-step process that can be divided into three major steps. First, eukaryotic translation initiation factor 2 (eIF2) binds with GTP and methionyl-tRNA to form the ternary complex, which further binds to 40S ribosomal subunit with the help of eIF1, eIF1A, eIF3 and eIF5 resulting the preinitiation complex (PIC). Second, the PIC binds to mRNA which is unwinded by the eIF4F complex (including eIF4E, eIF4G, eIF4A, eIF4B). Finally, the second GTPase, eIF5B catalyzes the joining of the 60S subunit and 40S subunit to form the 80S initiation complex (Jackson et al., 2010). mTORC1 regulates activities of a number of constituents of protein synthesis, including translation initiation factors and elongation factors, through phosphorylating two well-known substrates, the ribosomal S6 kinases (S6K1 and S6K2) and the eukaryotic initiation factor 4E binding proteins (4E-BP1 and 4E-BP2) (Gingras et al., 2001; Hay and Sonenberg, 2004). Phosphorylation of 4E-BP1 by mTORC1 increases the availability of eIF4E. Phosphorylation of eIF4B by S6K1 is necessary for PIC formation (Gingras et al., 2001). Ribosomal protein S6 (RPS6) which is the substrate of S6K1 and S6K2, involves in the regulation of cell proliferation, cell size and glucose homeostasis (Ruvinsky et al., 2005). However, systematic analysis of mTORC1 mediated phosphorylation in ribosomal and associated proteins has not been achieved.

To analyze mTORC1-mediated phosphorylation in ribosomal and associated proteins, quantitative phosphoproteomic analysis based on the SILAC (stable isotope labeling by amino acids in cell culture) method was carried out to analyze affinity enriched phosphoproteins from the untreated and rapamycin-treated 293T cells. The experimental workflow was displayed in Fig. 1A. Briefly, cells grown in light medium (^12^C_6_^14^N_2_-Lysine and ^12^C_6_-Arginine, K^0^R^0^) were treated with 200 nmol/L rapamycin for 2 h, while cells grown in heavy medium (^13^C_6_^15^N_2_-Lysine and ^13^C_6_-Arginine, K^8^R^6^) were untreated. Sucrose cushion centrifugation was used to isolate ribosomes. Proteins extracted from the whole cell lysate or the isolated ribosome fraction of the untreated and rapamycin-treated cells were mixed and trypsin digested. Then phosphopeptides were enriched with TiO_2_ beads and analyzed by nano-LC-MS/MS. The generated MS/MS spectra were searched against the human database using the Sequest search engine in the Proteome Discoverer (Version 1.4) software, with the false discovery rate (FDR) setting to 0.01.

**Figure 1:**
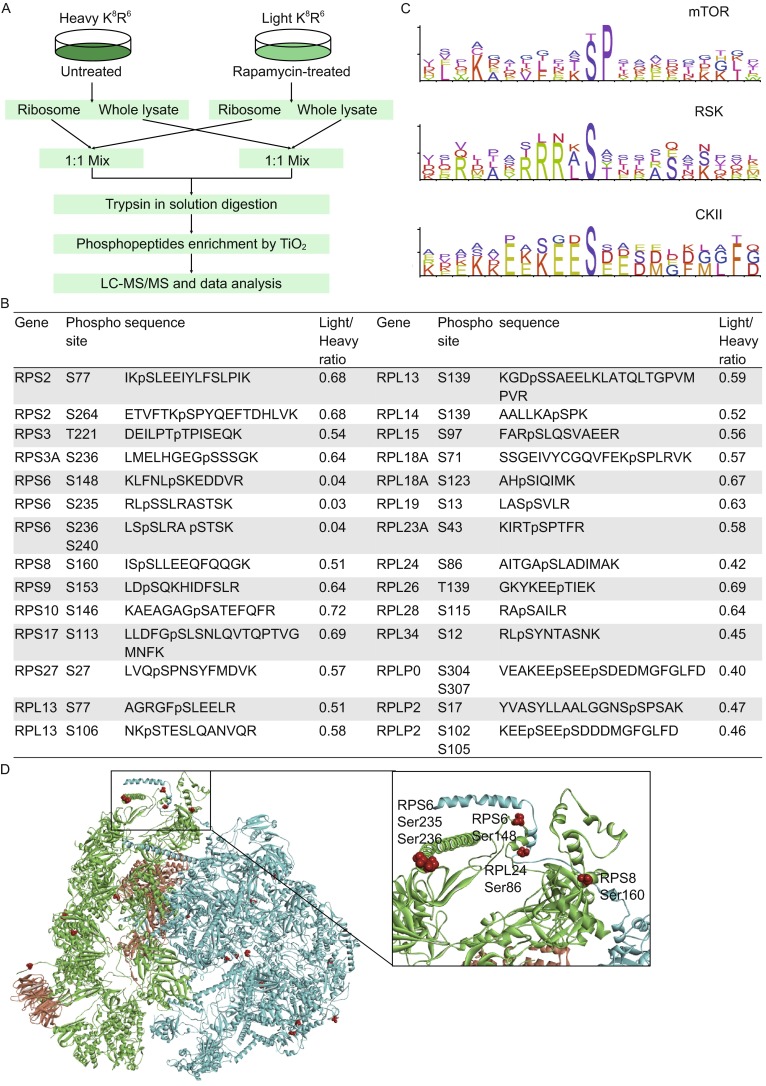
Phosphoproteomics analysis of ribosomal proteins after rapamycin treatment. (A) A schematic of the experimental procedure; (B) A list of mTOR-mediated phosphopeptides from ribosomal proteins; (C) Kinases and motifs analysis of mTOR-mediated phosphorylated peptides; (D) Locations of the mTOR-mediated phosphoresidues on human 80S ribosome. Proteins of the 40S subunit are shown in green, proteins of the 60S subunit are shown in blue, elongation factor 2 and guanine nucleotide-binding protein subunit beta-2-like 1 are shown in orange, and the mTOR-mediated phospho-residues are shown as red balls. The expanded view shows the location of phosphorylation sites on RPS6, RPS8 and RPL24

We identified 61 unique phosphorylation sites on 37 ribosomal proteins, among which 33 phosphorylation sites have been reported before in the Uniprot database (http://www.uniprot.org). Abundance of 28 phosphorylated peptides corresponding to 31 phosphorylation sites were decreased after rapamycin-treatment (Fig. 1B), of which 12 were from 9 proteins of the 40S ribosome, and 16 were from 12 proteins of the 60S ribosome. Quantitative proteomics using Tandem Mass Tags (TMT) was carried out and showed that 2-h rapamycin treatment did not induce changes in the expression levels of ribosomal proteins (Table S1). This indicated that phosphorylation changes in these proteins were caused by inhibition of mTOR. We further identified that 14 phosphorylated peptides possessed three types of consensus sequence motifs using iceLogo (http://iomics.ugent.be/icelogoserver/) (Fig. 1C and Table S2), including proline-directed motif (SP and TP), which was the known mTOR targeted phosphorylation sites, the ribosomal S6 kinase (RSK)-targeted arginine-rich motif (RRRxS), and the acidic motif (SxxD/E/pS) that was the potential substrate of casein kinase II (CKII) predicted using KinasePhos (http://kinasephos.mbc.nctu.edu.tw/).

Furthermore, out of the 31 mTORC1-mediated phosphorylation sites, 20 were on the surface of the 80S ribosome as highlighted with red color (Fig. 1D), which was downloaded from the Protein Data Bank (accession number: 4V6X). This suggests that mTOR and associated kinases facilely access and directly phosphorylate the 80S ribosome. Phosphorylation sites RPS6 Ser148, Ser235, Ser236, RPS8 Ser160 and RPL24 Ser86 were on the interface between 40S subunit and 60S subunit, indicating that mTOR mediated the interaction of 40S and 60S subunits. Phosphorylation of the C-terminal residues in RPS6 by mTOR is known to enhance the binding of 40S ribosome to the m7GpppG cap of mRNA for facile translation initiation (Hutchinson et al., 2011). Rapamycin-responsive RPS8 Ser160 and RPL24 Ser86 were in close proximity to the C-terminus of RPS6, suggesting mTOR-mediated RPS8 and RPL24 phosphorylation may also involve in mRNA binding. In summary, our phosphoproteomic results suggest that mTOR mediated the formation of the 80S ribosome and its binding with mRNA.

We identified 8353 unique phosphorylated peptides from 2761 distinct proteins extracted from the whole cell lysate. Abundance of ten phosphorylation peptides corresponding to eleven phosphorylation sites on seven translation initiation factors eIF2A, eIF3C, eIF3E, eIF4B, eIF4G1, eIF4G2 and eIF5B were decreased after rapamycin treatment (Fig. 2A and Table S3) and designated as mTOR regulated. eIF5B is responsible for the joining of 60S subunits with the pre-40S subunits and plays an important role in both of the cap-dependent and the internal ribosome entry site (IRES) dependent translation initiation (Jackson et al., 2010; Thakor and Holcik, 2012). In the present study, phosphorylation in four serine residues of eIF5B was decreased upon rapamycin treatment including phosphorylation on Ser214 residue, in consistent with the early report on mTOR or mitotic associated phosphoproteomics (Hsu et al., 2011; Kettenbach et al., 2011). A MS/MS spectrum that matched to the fragments of the peptide (NKPGPNIEpSGNEDDDASFK) containing phosphorylated Ser214 was shown in Fig. S1. Then we used the method of parallel reaction monitoring (PRM) based mass spectrometry combined with tandem mass tags (TMT) labeling to determine the intensity ratio of the phosphorylation peptide vs. the non-phosphopeptide and confirmed that the phosphorylation ratio was decreased from 0.3 to 0.1 upon rapamycin-treatment (Fig. S2).

**Figure 2:**
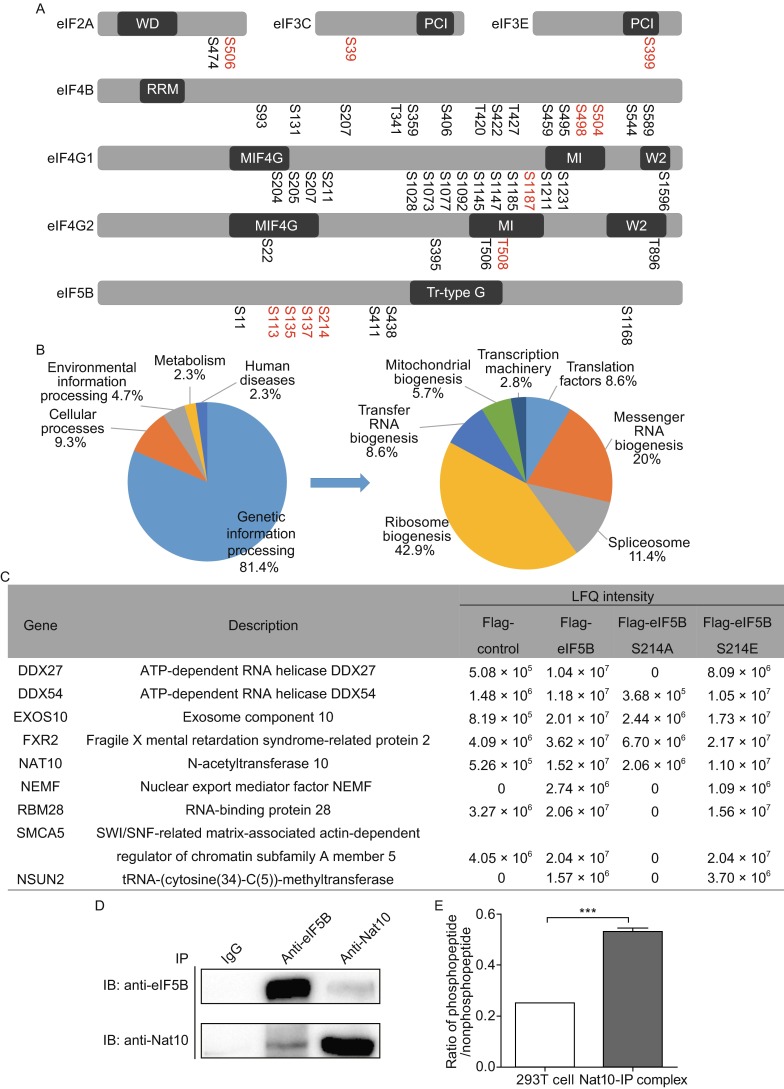
Analysis of mTOR-mediated phosphorylation changes in eukaryotic translation initiation factors. (A) Identified phosphorylation sites on translation initiation factors. The mTOR-mediated phosphorylation sites are red-coded. (B) GO analysis of the binding partners of the wild type eIF5B according to the associated biological processes; (C) A list of proteins preferentially binding to the eIF5B-S214E mutant; (D) Reciprocal immunoprecipitation of Nat10 and eIF5B; (E) The ratio of Ser214-containing phosphopeptide to the nonphosphopeptide determined by PRM-based MS analysis in eIF5B from the 293T cells and Nat10-immunoprecipiated complex. Data were analyzed using student’s *t* test. ***P < 0.001; *n* = 3

The mTOR-mediated phosphorylation in eIF2, eIF3, eIF4B and eIF4G has been reported before (Gingras et al., 2001; Hay and Sonenberg, 2004; Ozcan et al., 2008; Martineau et al., 2014). The present study also revealed that mTOR-inhibition decreases the phosphorylation in eIF5B. To explore effects of Ser214 phosphorylation on eIF5B functions, we used the CRISPR-cas9 technology to knockdown the expression of eIF5B, showing that the expression level of eIF5B in eIF5B-knockdown cells was one third of that in the control cells as determined by qPCR (Fig. S3). Then, plasmids containing the wildtype eIF5B, and two mutants eIF5B-S214A and eIF5B-S214E were transiently transfected into the eIF5B-knockdown cells, respectively, followed by immunoprecipitation to identify the binding partners of eIF5B and mutants. The mRNA level and protein expression of wild type eIF5B and two mutants were detected by qPCR, Western blotting and mass spectrometry, as shown in Figs. S3, S4 and S5. The binding partners of the wild type eIF5B and two mutants were identified by label free quantification (LFQ) using the MaxQuant software. When a protein was only present in the eIF5B immunoprecipitated complex or the total ion intensity of corresponding tryptic peptides from one protein identified in the eIF5B immunoprecipitated complex is five times higher than that in the FLAG-only immunoprecipitated complex, this protein was considered as the binding partner of eIF5B. Forty five binding partners (Table S4) of the wild type eIF5B were identified in two independent biological replicates and the interactome was analyzed using STRING software (http://string.embl.de) (Fig. S6). To understand the biological relevance of these proteins, the Gene Ontology (GO) was used to cluster proteins according to their associated biological processes. The annotations of gene lists were summarized via a pie plot based on KEGG pathway analysis as shown in Fig. 2B. Eighty percent of eIF5B-binding proteins participated in the genetic information processing, including ribosome biogenesis, messenger RNA biogenesis and spliceosome. In comparing to proteins binding to the wild type eIF5B, several proteins were found to preferentially bind to eIF5B-S214E mutant, but not eIF5B-S214A mutant as displayed in Fig. 2C. These proteins were considered to have the higher affinity to Ser214-phosphorylated eIF5B. Among them, N-acetyltransferase 10 (Nat10), a RNA acetyltransferase, is responsible for 18S rRNA processing through inducing N^4^-acetylcytidine formation (Ito et al., 2014). The Nat10-eIF5B interaction was verified by reciprocal immunoprecipitation (Fig. 2D) and eIF5B was immunoprecipitated by Nat10-specific antibody in Nat10-transfected 293T cells. Using PRM-based targeted mass spectrometry, we found that the ratio of the phosphorylated Ser214-containing peptide to the unphosphorylated peptide in the Nat10-immunoprecipitated eIF5B was 3 times higher than that in eIF5B from 293T cells, which confirmed that Nat10 preferred to bind to Ser214 phosphorylated eIF5B (Fig. 2E). It was reported that the mTOR pathway involved in rRNA maturation with unknown mechanism (Iadevaia et al., 2012). Our results suggested that mTOR phosphorylated eIF5B to enhance the binding of eIF5B with Nat10 and to promote rRNA processing.

Taken together, we applied quantitative phosphoproteomics to profile rapamycin-inhibition mediated phosphorylation changes in ribosomal proteins and suggested that mTOR mediated the 80S ribosome formation and its binding to mRNA. We further mapped mTOR-regulated phosphorylation sites in eukaryotic translation initiation factors and identified that phosphorylation of Ser214 on eIF5B enhanced the binding of eIF5B to the RNA-binding and processing proteins including Nat10. Our results suggest that the interaction of phosphorylated eIF5B with Nat10 promotes rRNA processing and rRNA maturation.

## FOOTNOTES

We thank the Protein Chemistry Facility at the Center for Biomedical Analysis of Tsinghua University for sample analysis. This work was supported in part by the National Natural Science Foundation of China (Grant No. 31270871 to H.T.D) and MOEC 2012Z02293 (H.T.D), the National Basic Research Program (973 Program) (No. 2014CBA02005 to H.T.D.) and the Global Science Alliance Program of Thermo-Fisher Scientific.

Xu Jiang, Shan Feng, Yuling Chen, Yun Feng and Haiteng Deng declare that they have no conflict of interest. This article does not contain any studies with human or animal subjects performed by the any of the authors.

## Electronic supplementary material

Below is the link to the electronic supplementary material.

Supplementary material 1 (DOCX 1259 kb)
