# Supplementary material for: Proteomic analysis of mTOR inhibition-mediated phosphorylation changes in ribosomal proteins and eukaryotic translation initiation factors
Source: Protein Cell. 2016 Jun 8;7(7):533–7. doi: 10.1007/s13238-016-0279-0 (PMC4930769; doi:10.1007/s13238-016-0279-0)
Supplement: Supplementary file 1 — Supplementary material 1 (DOCX 1259 kb) [file 13238_2016_279_MOESM1_ESM.docx]

Supplementary Table 1. The list of ribosomal proteins and eukaryotic translation initiation factors from rapamycin-treated and untreated cells.

| Accession | Gene | Discription | Score | Coverage | Rapamycin-treated/untreated |
| --- | --- | --- | --- | --- | --- |
| P15880 | RPS2 | 40S ribosomal protein S2 | 341.11 | 57.00 | 1.04 |
| P23396 | RPS3 | 40S ribosomal protein S3 | 1108.38 | 79.84 | 1.03 |
| P61247 | RPS3A | 40S ribosomal protein S3a | 596.05 | 72.35 | 1.03 |
| P62753 | RPS6 | 40S ribosomal protein S6 | 138.59 | 42.17 | 1.02 |
| P62241 | RPS8 | 40S ribosomal protein S8 | 331.75 | 52.88 | 1.02 |
| P46781 | RPS9 | 40S ribosomal protein S9 | 99.05 | 61.86 | 1.04 |
| P46783 | RPS10 | 40S ribosomal protein S10 | 189.69 | 50.30 | 1.09 |
| P08708 | RPS17 | 40S ribosomal protein S17 | 189.90 | 45.93 | 1.03 |
| P42677 | RPS27 | 40S ribosomal protein S27 | 122.23 | 41.67 | 1.04 |
| P26373 | RPL13 | 60S ribosomal protein L13 | 180.02 | 43.13 | 1.08 |
| E7EPB3 | RPL14 | 60S ribosomal protein L14 | 47.71 | 24.19 | 0.97 |
| P61313 | RPL15 | 60S ribosomal protein L15 | 282.96 | 53.92 | 1.07 |
| M0R3D6 | RPL18A | 60S ribosomal protein L18a | 192.85 | 46.10 | 1.12 |
| J3QR09 | RPL19 | 60S ribosomal protein L19 | 172.40 | 34.72 | 1.08 |
| P62750 | RPL23A | 60S ribosomal protein L23a | 231.20 | 53.21 | 1.06 |
| C9JXB8 | RPL24 | 60S ribosomal protein L24 | 77.73 | 52.07 | 1.04 |
| P61254 | RPL26 | 60S ribosomal protein L26 | 67.90 | 39.31 | 1.12 |
| P46779 | RPL28 | 60S ribosomal protein L28 | 112.26 | 45.99 | 1.11 |
| P49207 | RPL34 | 60S ribosomal protein L34 | 143.16 | 35.90 | 1.08 |
| P05388 | RPLP0 | 60S ribosomal protein P0 | 595.03 | 44.48 | 0.98 |
| P05387 | RPLP2 | 60S ribosomal protein P2 | 338.79 | 48.70 | 1.03 |
| Q9BY44 | EIF2A | Eukaryotic translation initiation factor 2A | 102.50 | 31.97 | 1.02 |
| H3BRV0 | EIF3C | Eukaryotic translation initiation factor 3 subunit C | 339.70 | 28.68 | 1.08 |
| P60228 | EIF3E | Eukaryotic translation initiation factor 3 subunit E | 222.86 | 34.61 | 1.00 |
| P23588 | EIF4B | Eukaryotic translation initiation factor 4B | 117.85 | 39.12 | 1.09 |
| Q04637-3 | EIF4G1 | Eukaryotic translation initiation factor 4 gamma 1 | 427.97 | 27.90 | 1.04 |
| H0Y3P2 | EIF4G2 | Eukaryotic translation initiation factor 4 gamma 2 | 147.39 | 24.05 | 1.05 |
| O60841 | EIF5B | Eukaryotic translation initiation factor 5B | 123.73 | 18.20 | 1.06 |

Supplementary Table 2. The list of mTOR-mediated phosphopeptides from ribosomal proteins.

| Gene | Discription | Phosphosite | Sequence | Light/Heavy ratio | Predicted kinase |
| --- | --- | --- | --- | --- | --- |
| RPS2 | 40S ribosomal protein S2 | S77 | IKpSLEEIYLFSLPIK | 0.68 | NA |
| RPS2 | 40S ribosomal protein S2 | S264 | ETVFTKpSPYQEFTDHLVK | 0.68 | mTOR |
| RPS3 | 40S ribosomal protein S3 | T221 | DEILPTpTPISEQK | 0.54 | mTOR |
| RPS3A | 40S ribosomal protein S3a | S236 | LMELHGEGpSSSGK | 0.64 | NA |
| RPS6 | 40S ribosomal protein S6 | S148 | KLFNLpSKEDDVR | 0.04 | NA |
| RPS6 | 40S ribosomal protein S6 | S235 | RLpSSLRASTSK | 0.03 | RSK |
| RPS6 | 40S ribosomal protein S6 | S236 | LSpSLRApSTSK | 0.04 | NA |
| RPS6 | 40S ribosomal protein S6 | S240 | LSpSLRApSTSK | 0.04 | RSK |
| RPS8 | 40S ribosomal protein S8 | S160 | ISpSLLEEQFQQGK | 0.51 | NA |
| RPS9 | 40S ribosomal protein S9 | S153 | LDpSQKHIDFSLR | 0.64 | ATM |
| RPS10 | 40S ribosomal protein S10 | S146 | KAEAGAGpSATEFQFR | 0.72 | NA |
| RPS17 | 40S ribosomal protein S17 | S113 | LLDFGpSLSNLQVTQPTVGMNFK | 0.69 | NA |
| RPS27 | 40S ribosomal protein S27 | S27 | LVQpSPNSYFMDVK | 0.57 | NA |
| RPL13 | 60S ribosomal protein L13 | S77 | AGRGFpSLEELR | 0.51 | NA |
| RPL13 | 60S ribosomal protein L13 | S106 | NKpSTESLQANVQR | 0.58 | RSK |
| RPL13 | 60S ribosomal protein L13 | S139 | KGDpSSAEELKLATQLTGPVMPVR | 0.59 | CKII |
| RPL14 | 60S ribosomal protein L14 | S139 | AALLKApSPK | 0.52 | mTOR |
| RPL15 | 60S ribosomal protein L15 | S97 | FARpSLQSVAEER | 0.56 | NA |
| RPL18A | 60S ribosomal protein L18a | S71 | SSGEIVYCGQVFEKpSPLRVK | 0.57 | mTOR |
| RPL18A | 60S ribosomal protein L18a | S123 | AHpSIQIMK | 0.67 | NA |
| RPL19 | 60S ribosomal protein L19 | S13 | LASpSVLR | 0.63 | NA |
| RPL23A | 60S ribosomal protein L23a | S43 | KIRTpSPTFR | 0.58 | mTOR |
| RPL24 | 60S ribosomal protein L24 | S86 | AITGApSLADIMAK | 0.42 | NA |
| RPL26 | 60S ribosomal protein L26 | T139 | GKYKEEpTIEK | 0.69 | NA |
| RPL28 | 60S ribosomal protein L28 | S115 | RApSAILR | 0.64 | RSK |
| RPL34 | 60S ribosomal protein L34 | S12 | RLpSYNTASNK | 0.45 | RSK |
| RPLP0 | 60S ribosomal protein P0 | S304 S307 | VEAKEEpSEEpSDEDMGFGLFD | 0.40 | CKII |
| RPLP2 | 60S ribosomal protein P2 | S17 | YVASYLLAALGGNSpSPSAK | 0.47 | mTOR |
| RPLP2 | 60S ribosomal protein P2 | S102 S105 | KEEpSEEpSDDDMGFGLFD | 0.46 | CKII |

Supplementary Table 3. The list of mTOR-mediated phosphopeptides from eukaryotic translation initiation factors.

| Gene | Discription | Phosphosite | Sequence | Light/Heavy ratio |
| --- | --- | --- | --- | --- |
| EIF2A | Eukaryotic translation initiation factor 2A | S506 | SDKpSPDLAPTPAPQSTPR | 0.66 |
| EIF3C | Eukaryotic translation initiation factor 3 subunit C | S39 | QPLLLpSEDEEDTKR | 0.71 |
| EIF3E | Eukaryotic translation initiation factor 3 subunit E | S399 | LGHVVMGNNAVpSPYQQVIEK | 0.30 |
| EIF4B | Eukaryotic translation initiation factor 4B | S498 | SQSpSDTEQQpSPTSGGGk | 0.48 |
| EIF4B | Eukaryotic translation initiation factor 4B | S504 | SQSpSDTEQQpSPTSGGGk | 0.48 |
| EIF4G1 | Eukaryotic translation initiation factor 4 gamma 1 | S1187 | SFpSKEVEER | 0.20 |
| EIF4G2 | Eukaryotic translation initiation factor 4 gamma 2 | T508 | TQpTPPLGQTPQLGLK | 0.50 |
| EIF5B | Eukaryotic translation initiation factor 5B | S113 | QSFDDNDpSEELEDKDSK | 0.37 |
| EIF5B | Eukaryotic translation initiation factor 5B | S135 S137 | VEMYpSGpSDDDDDFNKLPK | 0.50 |
| EIF5B | Eukaryotic translation initiation factor 5B | S214 | NKPGPNIEpSGNEDDDASFK | 0.67 |

Supplementary Table 4. The list of binding partners of wildtype eIF5B and two mutants (eIF5B-S214A and eIF5B-S214E).

| Gene | Discription | LFQ intensity | | | |
| --- | --- | --- | --- | --- | --- |
|  |  | **Flag-control** | **Flag-eIF5B** | **Flag-eIF5B S214A** | **Flag-eIF5B S214E** |
| ATAD3A | ATPase family AAA domain-containing protein 3A | 0 | 2.05E+06 | 1.84E+06 | 7.40E+05 |
| DDX18 | ATP-dependent RNA helicase DDX18 | 3.44E+06 | 3.63E+07 | 2.18E+07 | 2.13E+07 |
| DDX27 | ATP-dependent RNA helicase DDX27 | 5.08E+05 | 1.04E+07 | 0 | 8.09E+06 |
| DDX50 | ATP-dependent RNA helicase DDX50 | 4.83E+05 | 1.15E+07 | 2.26E+06 | 6.62E+06 |
| DDX54 | ATP-dependent RNA helicase DDX54 | 1.48E+06 | 1.18E+07 | 3.68E+05 | 1.05E+07 |
| CKMT1A | Creatine kinase U-type, mitochondrial | 0 | 7.84E+05 | 4.08E+05 | 0 |
| POLRMT | DNA-directed RNA polymerase, mitochondrial | 2.45E+06 | 1.83E+07 | 2.10E+07 | 1.95E+07 |
| STAU1 | Double-stranded RNA-binding protein Staufen homolog 1 | 6.45E+05 | 5.50E+06 | 4.01E+06 | 1.18E+06 |
| TRIM25 | E3 ubiquitin/ISG15 ligase TRIM25 | 4.53E+05 | 1.21E+07 | 2.35E+06 | 2.26E+06 |
| EIF2S2 | Eukaryotic translation initiation factor 2 subunit 2 | 2.44E+07 | 1.27E+08 | 1.40E+08 | 5.18E+07 |
| EIF2S3 | Eukaryotic translation initiation factor 2 subunit 3 | 2.69E+07 | 1.81E+08 | 1.41E+08 | 9.80E+07 |
| EIF5 | Eukaryotic translation initiation factor 5 | 0 | 5.05E+07 | 1.95E+07 | 3.08E+06 |
| EXOS6 | Exosome complex component MTR3 | 1.25E+06 | 7.84E+06 | 1.09E+07 | 3.99E+06 |
| EXOS10 | Exosome component 10 | 8.19E+05 | 2.01E+07 | 2.44E+06 | 1.73E+07 |
| FMR1 | Fragile X mental retardation protein 1 | 0 | 1.90E+06 | 3.59E+05 | 0 |
| FXR1 | Fragile X mental retardation syndrome-related protein 1 | 1.07E+07 | 7.06E+07 | 3.87E+07 | 4.46E+07 |
| FXR2 | Fragile X mental retardation syndrome-related protein 2 | 4.09E+06 | 3.62E+07 | 6.70E+06 | 2.17E+07 |
| GRWD1 | Glutamate-rich WD repeat-containing protein 1 | 8.29E+05 | 6.85E+06 | 6.42E+06 | 1.60E+06 |
| GNL3 | Guanine nucleotide-binding protein-like 3 | 2.37E+06 | 1.39E+07 | 1.01E+07 | 1.51E+07 |
| DKC1 | H/ACA ribonucleoprotein complex subunit 4 | 0 | 1.57E+06 | 3.14E+06 | 0 |
| HP1BP3 | Heterochromatin protein 1-binding protein 3 | 0 | 5.93E+06 | 3.26E+06 | 4.98E+06 |
| HNRNPD | Heterogeneous nuclear ribonucleoprotein D0 | 0 | 4.75E+05 | 0 | 0 |
| HMMR | Hyaluronan mediated motility receptor | 0 | 4.09E+05 | 4.90E+05 | 6.53E+05 |
| NAT10 | N-acetyltransferase 10 | 5.26E+05 | 1.52E+07 | 2.06E+06 | 1.10E+07 |
| NONO | Non-POU domain-containing octamer-binding protein | 0 | 7.38E+05 | 1.42E+06 | 0 |
| NEMF | Nuclear export mediator factor NEMF | 0 | 2.74E+06 | 0 | 1.09E+06 |
| YBX1 | Nucleolar GTP-binding protein 1 | 3.24E+06 | 1.64E+07 | 1.38E+07 | 1.31E+07 |
| NOP56 | Nucleolar protein 56 | 2.79E+06 | 1.59E+07 | 8.59E+06 | 6.16E+06 |
| NOP58 | Nucleolar protein 58 | 1.53E+06 | 7.72E+06 | 4.45E+06 | 3.53E+06 |
| PES1 | Pescadillo homolog | 0 | 5.13E+06 | 2.48E+06 | 2.16E+06 |
| FARSB | Phenylalanine--tRNA ligase beta subunit | 0 | 1.44E+06 | 2.51E+05 | 2.03E+05 |
| SERBP1 | Plasminogen activator inhibitor 1 RNA-binding protein | 0 | 4.27E+05 | 0 | 0 |
| PA2G4 | Proliferation-associated protein 2G4 | 1.06E+06 | 7.00E+06 | 1.36E+07 | 2.72E+06 |
| IQGAP1 | Ras GTPase-activating protein-binding protein 1 | 1.20E+06 | 6.63E+06 | 4.39E+06 | 0 |
| UPF1 | Regulator of nonsense transcripts 3B | 0 | 1.15E+06 | 1.94E+06 | 0 |
| RSL1D1 | Ribosomal L1 domain-containing protein 1 | 3.14E+06 | 1.57E+07 | 2.15E+07 | 1.54E+07 |
| RRP1B | Ribosomal RNA processing protein 1 homolog B | 5.62E+05 | 5.94E+06 | 2.45E+06 | 1.34E+06 |
| RBM28 | RNA-binding protein 28 | 3.27E+06 | 2.06E+07 | 0 | 1.56E+07 |
| SRP68 | Signal recognition particle subunit SRP68 | 7.13E+05 | 5.59E+06 | 3.68E+06 | 5.46E+06 |
| SRP72 | Signal recognition particle subunit SRP72 | 9.42E+05 | 5.69E+06 | 5.10E+06 | 3.57E+06 |
| SND1 | Staphylococcal nuclease domain-containing protein 1 | 0 | 1.54E+06 | 0 | 5.91E+05 |
| SMCA5 | SWI/SNF-related matrix-associated actin-dependent regulator of chromatin subfamily A member 5 | 4.05E+06 | 2.04E+07 | 0 | 2.04E+07 |
| NSUN2 | tRNA (cytosine(34)-C(5))-methyltransferase | 0 | 1.57E+06 | 0 | 3.70E+06 |
| RTCB | tRNA-splicing ligase RtcB homolog | 0 | 2.48E+06 | 2.21E+06 | 0 |
| TUBB6 | Tubulin beta-6 chain | 0 | 3.69E+06 | 2.41E+06 | 1.24E+06 |


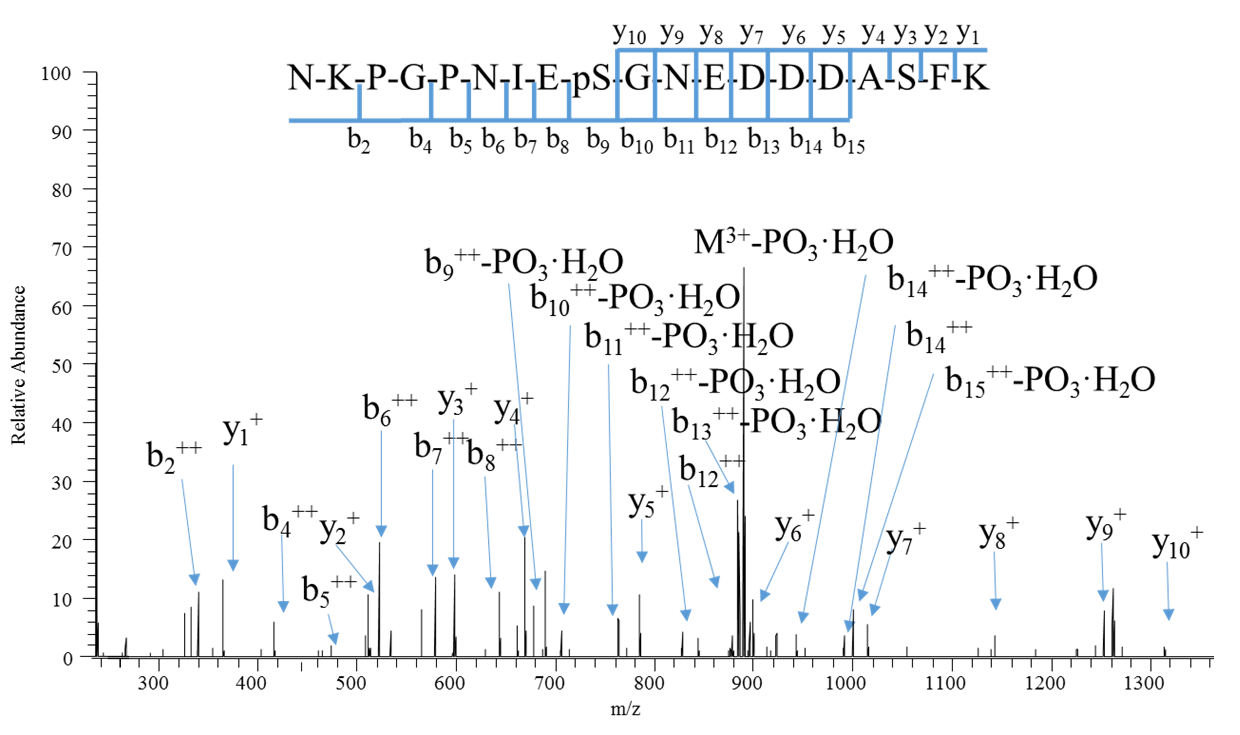


Supplementary Figure 1. A MS/MS spectrum of the peptide containing phosphorylated Ser214 of eIF5B.


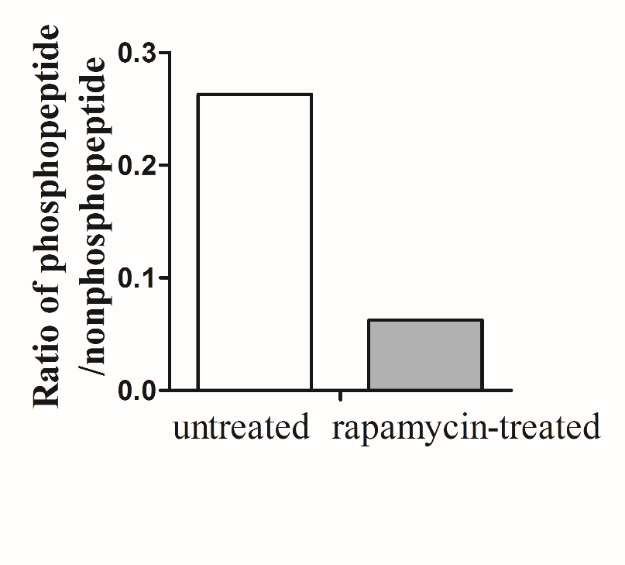


Supplementary Figure 2. The ratio of the phosphorylated Ser214-containing peptide to unphosphorylated peptide from untreated and rapamycin-treated samples.


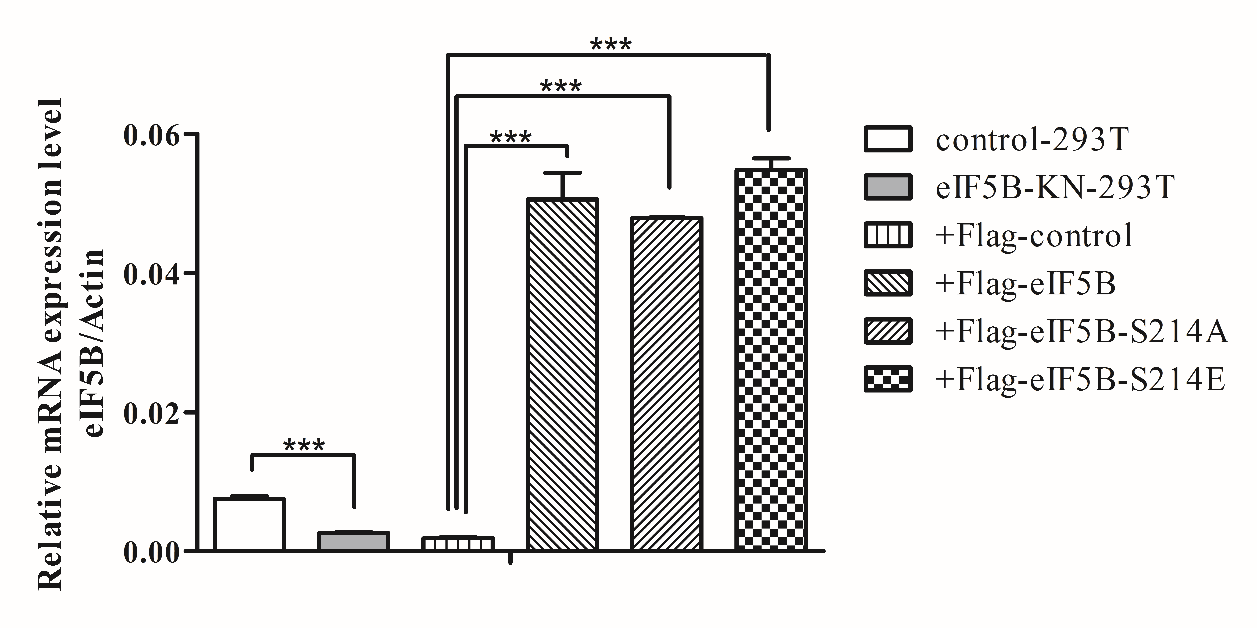


Supplementary Figure 3. The qPCR analysis of eIF5B mRNA expression levels in eIF5B-knockdown cells and the eIF5B, eIF5B-S214A and eIF5B-S214E overexpression cells.


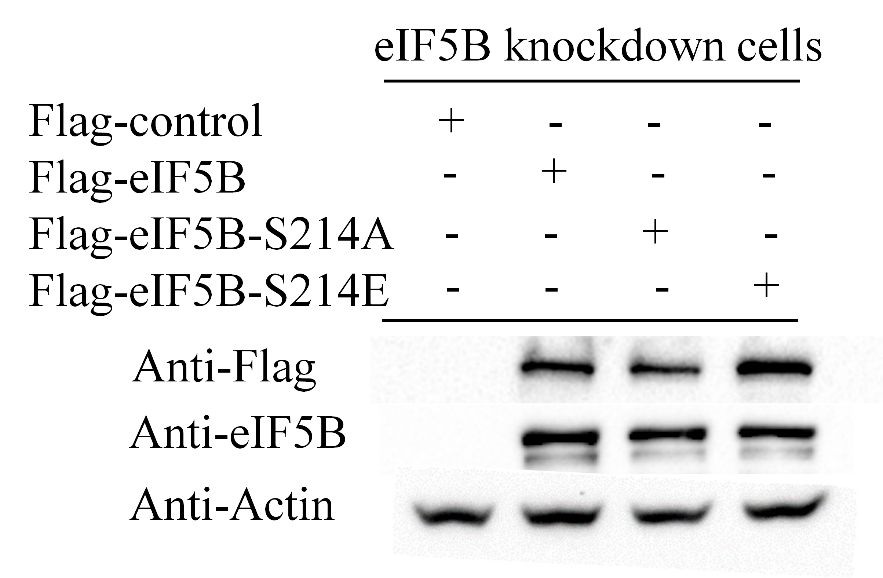


Supplementary Figure 4. Western blotting of eIF5B in eIF5B knockdown cells and the eIF5B, eIF5B-S214A and eIF5B-S214E overexpression cells.


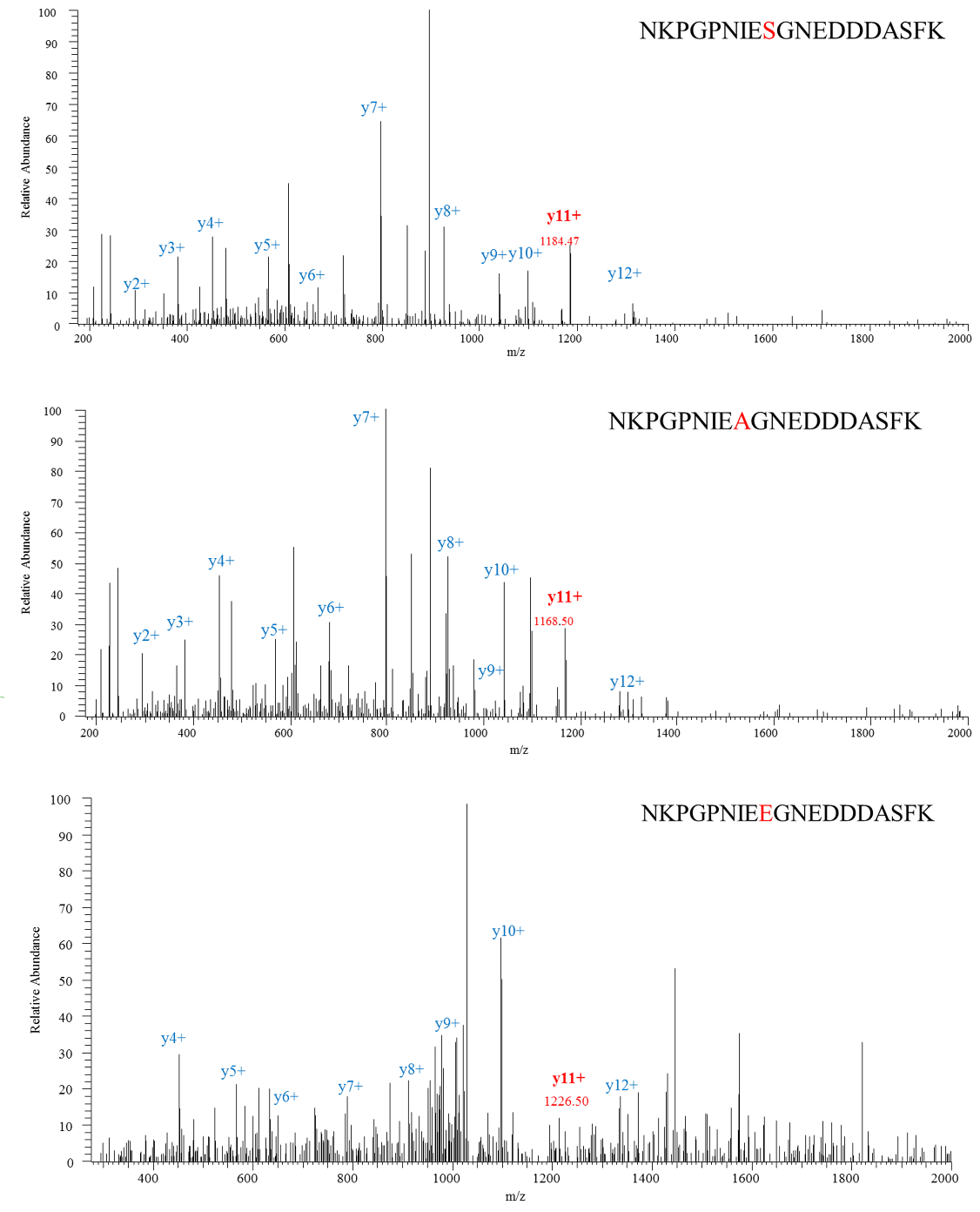


Supplementary Figure 5. The MS/MS spectra of the Ser214-, Ala214- and Glu214- containing peptide of eIF5B from the eIF5B, eIF5B-S214A and eIF5B-S214E overexpression cells.


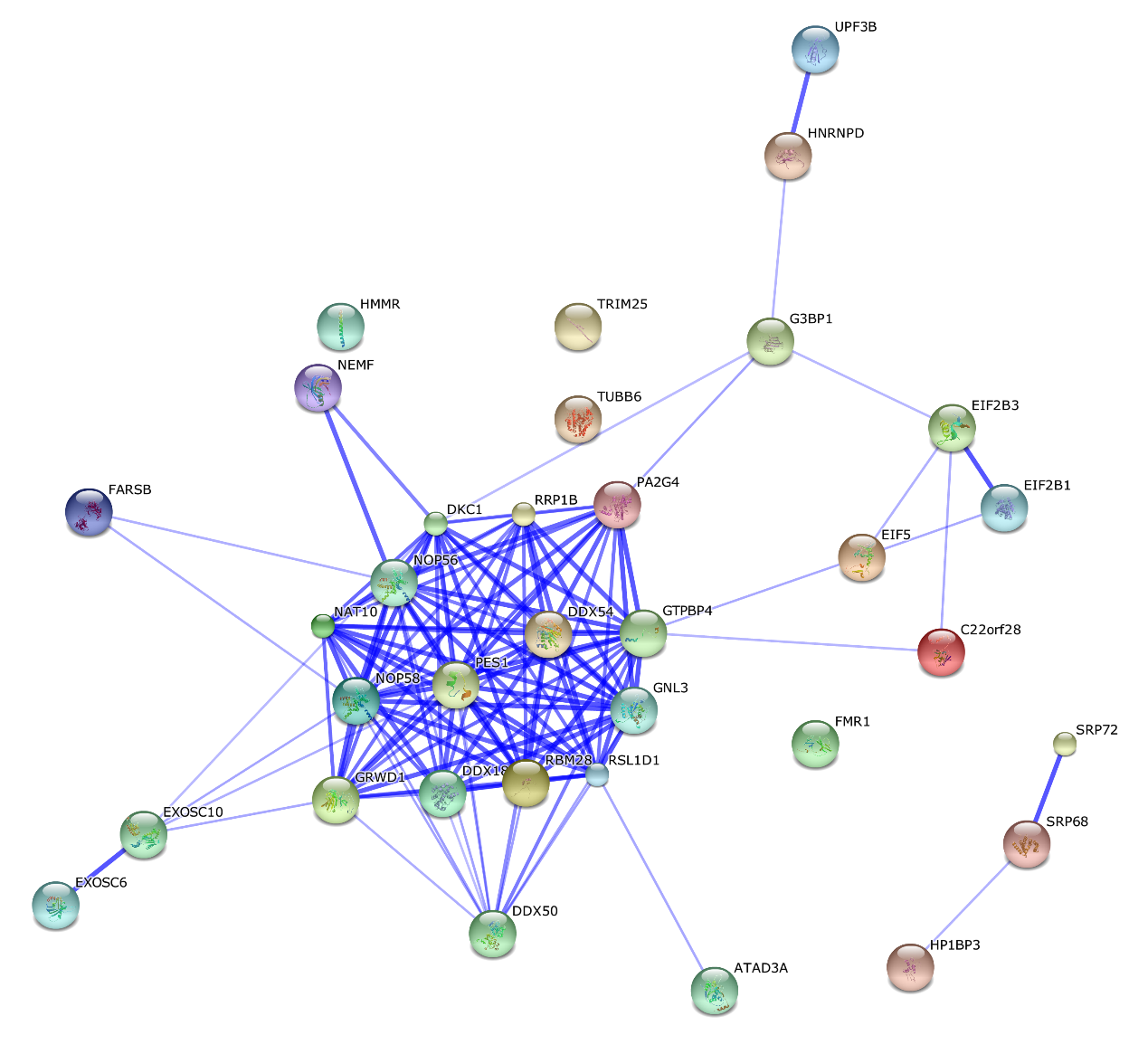


Supplementary Figure 6. Interactome analysis using the STRING software.

**Materials and Methods**

**Chemicals and Reagents**

Dulbecco’s modified Eagle medium (DMEM), normal and dialyzed fetal bovine serum (FBS and D-FBS) and penicillin/streptomycin were purchased from Wisent (Saint-Jean-Baptiste, CA). SILAC DMEM medium, isotope labeling ^13^C_6_^15^N_2_-Lysine, ^13^C_6_-Arginine, ^12^C_6_^14^N_2_-Lysine, ^12^C_6_-Arginine and protein A/G agarose were purchased from Thermo (Waltham, MA). Dithiothreitol (DTT) and BCA protein assay kit were purchased from Solarbio (Beijing, China). Iodoacetamide (IAA) and the Flag M2 Affinity Gel were purchased from Sigma (St Louis, MO). Sequencing grade trypsin was purchased from Promega (Fitchburg, WI). TiO_2_ beads were purchased from GL Sciences (Tokyo, Japan). The TMT labeling reagent was purchased from Thermo-Pierce Biotechnology (Rockford, IL). Anti-eIF5B and anti-Nat10 antibodies were purchased from Proteintech (Wuhan, China). Anti-actin antibody was purchased from Abmart (Shanghai, China). Anti-mouse and anti-rabbit secondary antibodies were purchased from Cell Signaling Technology (Boston, MA). The Total RNA Isolation System and Reverse Transcription kit was purchased from TIANGEN (Beijing, China).

**Cell Culture and SILAC Labeling**

Human embryonic kidney 293T cell line was obtained from the cell bank of the Chinese Academy of Sciences (Shanghai, China). Cells were grown in DMEM medium supplemented with 10% FBS and 1% penicillin/streptomycin at 37°C with 5% CO_2_. For SILAC labeling, cells were culture in SILAC culture medium, which was made by mixing SILAC DMEM medium with 10% D-FBS, 1% penicillin/streptomycin, 146 mg/L isotope labeling ^13^C_6_^15^N_2_-Lysine, and 84 mg/L ^13^C_6_-Arginine. 293T cells were grown for 10 passages in SILAC medium and all proteins were fully incorporated.

**Sample Preparation and Ribosome isolation**

Cells grown in light medium (^12^C_6_^14^N_2_-Lysine and ^12^C_6_-Arginine, K^0^R^0^) were treated with 200 nM rapamycin for 2 hours, while cells grown in heavy medium (^13^C_6_^15^N_2_-Lysine and ^13^C_6_-Arginine, K^8^R^6^) were untreated. Cells were washed twice with PBS and lysed using 8 M urea in PBS. The whole cell lysate was centrifuged at 14,000 × g for 30 minutes at 4°C. For ribosome isolation, cells were lysed in ice-cold extraction buffer (50 mM Tris–HCl, 100 mM KCl, 5 mM MgCl_2_, 0.7% NP-40, 0.1 mg/ml cycloheximide, 1 mM DTT, protease inhibitor and phosphatase inhibitor). Cell lysates were centrifuged at 17 000 × g at 4°C for 20 minutes to remove nuclei, mitochondria and cell debris. The supernatant was loaded on 1 M sucrose cushion prepared in extraction buffer and centrifuged at 250 000 × g at 4°C for 4 hours (Beckman Optima MAX-XP, TLA-100.3 rotor). The ribosomal pellet was washed with extraction buffer and resuspended in 8 M urea in PBS. Protein concentrations were determined with the BCA method.

**Quantitative Phosphoproteomic Analysis**

Equal amounts of proteins from the whole lysate or the ribosomal fraction from untreated and rapamycin-treated cells were mixed, digested using trypsin, desalted with Sep-Pak C18 Vac cartridges (Waters, Milford, MA) and fractionated by off-line high-pH chromatography (HpH) before subsequent titanium dioxide (TiO_2_) enrichment and LC-MS/MS analysis (Batth et al., 2014). The generated MS/MS spectra were searched using a Sequest HT Algorithm of Proteome Discoverer software (version 1.4, Thermo Scientific, USA). In the search criteria, SILAC ^13^C_6_^15^N_2_-Lysine and ^13^C_6_-Arginine (+8.014 Da at lysine and +6.020 Da at arginine) and phosphorylation (STY) were set as the variable modification and two missed cleavages were allowed. The PhosphoRS algorithm was used to calculate the probability of the phosphorylation sites. When the PhosphoRS probability was above 75%, the phosphorylation site was considered to be true. The experiment was carried out in two biological replicates.

**Protein Quantitation by Parallel Reaction Monitoring (PRM)**

PRM is a method to quantify multiple targeted peptides with the high sensitivity and specificity, in which the target precursor ion is isolated by the quadrupole mass filter and the fragment ions are detected in the Orbitrap mass analyzer. In the present experiment, equal amounts of proteins from untreated and rapamycin-treated cells were separated by 1D SDS-PAGE, respectively. The gel bands of eIF5B were excised and digested as previously described (Hu et al., 2014). Peptides from untreated cells labeled by TMT^6^-127 and peptides from rapamycin-treated cells labeled by TMT^6^-128 were mixed. The targeted peptides were synthesized and added as standard. 5 ng nonphosphorylated peptide (NKPGPNIESGNEDDDASFK) and 1 ng phosphorylated peptide (NKPGPNIEpSGNEDDDASFK) were labeled by TMT^6^-126, added into the mixture and analyzed by Thermo Scientific Q Exactive mass spectrometer in PRM mode. The PRM analysis was performed with the resolution of 17,500, the target AGC values of 1 × 10^6^, individual isolation window of 2 Th window and maximum fill times within 100 ms. Fragmentation was performed with a normalized collision energy of 25. The absolute amount of the targeted peptide was quantified with the help of the standard peptide. Then the ratio of the phosphopeptide vs the nonphosphopeptide was calculated. This method was also used to detect the phosphorylation ratio of the Nat10-immunoprecipated eIF5B and the eIF5B from 293T cells.

**Establishment of eIF5B knockdown cells**

The CRISPR/cas9 technology was used to knockdown the eIF5B gene in 293T cells as previously reported (Ran et al., 2013). The eIF5B-specific gRNA sequence was 5’-GAGCGCCATTGACAAGCAATGGG-3’ and non-silencing scrambled gRNA sequence was 5’-ACGATACAAGGCTGTTAGAGAG-3’. The clone with decreased eIF5B expression was selected as the eIF5B knockdown cells.

**Isolation of the binding partners of the wild type eIF5B, eIF5B-S214A and eIF5B-S214E**

The human eIF5B cDNA was synthesized from the total RNA of 293T cells. The recombinant eIF5B with flag tag was cloned into the plasmid pcDNA3.1 to create the pcDNA3.1-Flag-eIF5B vector. Then the Site-Directed Mutagenesis Kit (Thermo Scientific, USA) was used to generate two mutant plasmids: pcDNA3.1-Flag-eIF5B-S214A and pcDNA3.1-Flag-eIF5B-S214E according to the manufacturer’s instruction.

Transient transfections of plasmids containing the Flag-only, Flag-eIF5B, Flag-eIF5B-S214A and Flag-eIF5B-S214E were performed with Lipofectamine 2000 from Invitrogen according to the manufacturer’s instruction. Cells were harvested after 48 hours and lysed in lysis buffer (50 mM Tris-HCl pH 7.5, 150 mM NaCl, 1% Triton X-100, 1% sodium pyrophosphate, protease inhibitor and phosphatase inhibitor) at 4°C for 30 minutes. The supernatants collected by centrifugation at 14,000×g at 4°C for 20 minutes were incubated with the Flag M2 Affinity Gel at 4°C for 4 hours. After incubation, the gels were washed four times with lysis buffer and bound proteins were eluted with SDS-PAGE sample buffer.

**Protein separation by 1D SDS-PAGE and mass spectrometry analysis**

The samples of isolated complexes were separated by 1D SDS-PAGE, digested with trypsin and analyzed using an LTQ-Orbitrap mass spectrometer as previously described (Hu et al., 2014). The generated MS/MS spectra were searched against the human.fasta database downloaded from Uniprot using the MaxQuant software. All proteins were taken for label free quantification (LFQ) analysis. Through comparing the LFQ intensity of eIF5B immunoprecipitated complex with the FLAG-only immunoprecipitated complex, the binding partners of eIF5B were identified.

**Immunoprecipitation**

Equal amounts of 293T cell lysates were incubated at 4 °C overnight with 10 µl anti-eIF5B antibody, 10 µl anti-Nat10 antibody or without antibody, respectively, followed by 2 hours incubation with 30 μl 50% (v/v) protein A/G agarose. Pellets were washed five times in RIPA buffer and proteins were eluted with SDS-PAGE sample buffer. Then immumoblot analysis was carried out with the eIF5B and Nat10 antibodies. The Nat10-immumoprecipiated complex was also separated by 1D SDS-PAGE and analyzed by the PRM-based MS as described above.

**Real-Time Quantitative PCR (qPCR) and Western Blotting**

The expression of eIF5B in cells was detected using qPCR and western blotting as previously described (Hu et al., 2014). The primers of eIF5B using in qPCR analysis were listed as follows: sense strand 5’- TGAAGGCTTCAGTGATGTTGGA -3’ and antisense strand 5’- AACTCCTAAACTATCAGCCATTTCTTGT -3’.

**Statistical Method**

Statistical analysis was performed with GraphPad Prism 5.0 software by using Student’s t test. The *P* values less than 0.05 were considered statistically significant.

Reference

Batth, T.S., Francavilla, C., and Olsen, J.V. (2014). Off-line high-pH reversed-phase fractionation for in-depth phosphoproteomics. J Proteome Res 13, 6176-6186.

Hu, Y., Wang, H., Wang, Q., and Deng, H. (2014). Overexpression of CD38 decreases cellular NAD levels and alters the expression of proteins involved in energy metabolism and antioxidant defense. J Proteome Res 13, 786-795.

Ran, F.A., Hsu, P.D., Wright, J., Agarwala, V., Scott, D.A., and Zhang, F. (2013). Genome engineering using the CRISPR-Cas9 system. Nature Protocols 8, 2281-2308.
